# Preferences for treatment for latent tuberculosis infection in primary care among people in the USA at increased risk of tuberculosis: a pilot survey

**DOI:** 10.1136/bmjopen-2026-121108

**Published:** 2026-10-14

**Authors:** Hélène E Aschmann, Amy S Tang, Meagan Lee, Katya L Salcedo, Matthew T Murrill, Gina Chen, Yuqian Ouyang, Kit Lui, Mehabuba Rahman, Jennifer Flood, Andrew D Kerkhoff, Tracy K Lin, Priya B Shete, for the Tuberculosis Epidemiologic Studies Consortium

**Affiliations:** 1 Division of Pulmonary and Critical Care Medicine, Zuckerberg San Francisco General Hospital and Trauma Center, University of California San Francisco, San Francisco, California, USA; 2 Center for Tuberculosis, University of California San Francisco, San Francisco, California, USA; 3 North East Medical Services, San Francisco, California, USA; 4 Institute for Global Health Sciences, University of California San Francisco, San Francisco, California, USA; 5 Tuberculosis Control Branch, California Department of Public Health, Richmond, California, USA; 6 Division of Hospital Medicine, University of California San Francisco, San Francisco, California, USA; 7 Division of Tuberculosis Elimination, National Center for HIV, Viral Hepatitis, STD, and TB Prevention, Centers for Disease Control and Prevention, Atlanta, Georgia, USA; 8 Division of HIV, Infectious Diseases and Global Medicine, Zuckerberg San Francisco General Hospital and Trauma Center, University of California San Francisco, San Francisco, California, USA; 9 Department of Social and Behavioral Sciences, Institute for Health & Aging, University of California San Francisco, San Francisco, California, USA

**Keywords:** Tuberculosis, Patient Preference, Person-Centered Care, PREVENTIVE MEDICINE, Primary Health Care, PUBLIC HEALTH

## Abstract

**Abstract:**

**Objectives:**

Tuberculosis (TB) in the USA disproportionately affects non-US-born individuals. While testing this population for TB infection is recommended, little is known about individuals’ willingness to take treatment for latent TB infection (LTBI). To understand which factors and perspectives around TB and LTBI treatment most influence individuals’ willingness to take LTBI treatment, we conducted a pilot preference survey among individuals from countries with high TB incidence.

**Design:**

Cross-sectional survey supported by language concordant community health workers.

**Setting:**

Consecutive recruitment at a federally qualified health centre, serving a primarily Asian immigrant community, in San Francisco.

**Participants:**

Adults seeking primary care, eligible for risk-based LTBI testing based on place of birth.

**Outcome measures:**

Perspectives on TB disease, risk of reinfection, and willingness to accept treatment if diagnosed with LTBI conditional on different factors, such as side effects, costs and other treatment burden.

**Results:**

Among 60 participants, the median age was 48 years (IQR 35–63 years), 52% were women and 100% spoke Chinese. Infecting others (n=35, 58%), risk of death (n=30, 50%) and potential isolation (n=25, 42%) were the most worrisome consequences of TB disease. Reinfection risk, risk of liver damage, cost, TB progression risk, clinic visits and blood draws were most often rated moderately or very important when considering LTBI treatment (n=53 to 57, 88–95%). While most participants (n=56, 93%) were willing to take treatment if diagnosed with LTBI even at a 10-year TB progression risk below 1% and willing to accept a risk of liver damage (n=41, 68%), less than half would accept LTBI treatment if there were any associated cost (n=28, 47%). Finally, many participants had concerns about their reinfection risk after completing LTBI treatment (n=34, 57%).

**Conclusions:**

Among surveyed participants, TB disease and its consequences such as hospitalisation, death and infecting others were worrisome, and participants had a high level of willingness to take treatment if diagnosed with LTBI. Future assessments of how people weigh trade-offs regarding LTBI diagnosis and treatment could inform interventions to increase LTBI treatment acceptance and completion.

**STRENGTHS AND LIMITATIONS OF THIS STUDY**

- This pilot preference study had a high completion rate, included a detailed survey and met the pre-specified precision required to guide the design and selection of attributes and levels of a future choice-based study.
- The survey was administered by language-concordant community health workers, enabling broad inclusion of participants across age and education levels within a population with a high prevalence of latent TB infection (LTBI).
- Administration by community health workers may have introduced some social desirability bias.
- The study describes hypothetical choices and was not designed to measure trade-offs.
- Participants are not representative of all non-US-born populations.

## Introduction

 To achieve tuberculosis (TB) elimination in the USA, broad populations must be treated for latent TB infection (LTBI).^1^ People with LTBI are infected with *Mycobacterium tuberculosis* and do not have symptoms, but are at risk of progressing to TB disease. Although the overall TB incidence rate in the USA is low (2.9 per 100 000 persons in 2023), non-US-born communities are disproportionately affected by LTBI, with 76% of TB diagnoses in the USA occurring among these communities.^2^ Among non-US-born Asian persons, the TB incidence rate is nearly eight times the overall national rate (22.5 per 100 000 persons).^2^ LTBI treatment is effective at preventing TB disease. Despite recommendations to increase testing and treatment for LTBI among persons with risk factors,^3^ large gaps persist in the TB preventive care cascade.^4^ Steps in the LTBI care cascade with greater losses include treatment recommendation by providers and treatment completion.^4^ Patient-level barriers to TB preventive care include limited healthcare access, lack of insurance coverage, transportation challenges, economic factors, fear of deportation and low perceived risk of TB.^4 5^ Provider-level barriers can include time pressures during clinic visits and knowledge gaps in how to test for and treat LTBI.^4 6 7^ At the health system-level, competing priorities, chronic underfunding of primary care and long wait times further hamper TB prevention.^4 8^ To reduce these gaps and understand whether public health goals of eliminating TB are feasible and compatible with individual decisions among people at risk of progressing to TB,^1^ it is crucial to understand under which conditions individuals would accept therapy for LTBI. Although some preference studies compared regimen features in diverse settings,^9–12^ little is known on how characteristics related to persons influence preferences for accepting LTBI treatment, such as presumed progression risk or whether the person would need to modify or stop a current medication.

Studies among migrants have identified multiple barriers for LTBI treatment initiation and adherence.^13–15^ These barriers represent challenges that migrants may perceive regarding LTBI treatment and may affect uptake. These challenges include taking pills, the duration of treatment, clinic visits and blood tests, as well as changes to other medications due to drug–drug interactions with rifamycins, willingness to cease alcohol consumption during the treatment and potential costs of LTBI care, including loss of income and opportunity costs.^13 16 17^ In addition, a challenge may be the risk of reinfection after successful treatment, as this may limit the benefits of LTBI treatment in time (ie, preventing TB and its consequences). This may matter particularly to individuals planning to travel to countries with higher TB incidence.^18^ On the other hand, individuals may also perceive the benefits of LTBI treatment. Beyond reducing the risk of progressing to TB, LTBI treatment helps avoid the consequences of TB, including increased risk of death,^19^ hospitalisation,^20 21^ significant symptoms, anticipated stigma, isolation and lengthy treatment regimens of at least 4 or 6 months.^22^ Finally, many TB survivors experience post-TB lung disease, reducing quality of life long-term and increasing mortality.^23^

To assess which of these challenges and benefits exert the most influence on LTBI treatment-related decisions, we conducted a pilot survey of non-US-born persons eligible for LTBI testing. The survey (1) elicited which explanations of TB and its consequences resonated most with participants and (2) assessed how different factors potentially affect the acceptability of LTBI treatment. These findings will inform the development of a large, choice-based survey to precisely quantify trade-offs individuals are willing to accept for LTBI treatment to inform clinical practice for primary care and person-centred interventions (eg, decision-making tools or provider education) to address gaps in the LTBI cascade of care.

## Methods

### Setting and participants

We conducted a cross-sectional pilot survey among individuals eligible for LTBI testing in October 2023. The study took place at one of the San Francisco clinic locations of North East Medical Services, a federally qualified community health centre serving a large low-income Asian immigrant community.^24^ Individuals were eligible if they were born in a country with intermediate or high incidence of TB^25^ (incidence>10 per 100 000 persons), aged 18 or older, had no known prior history of TB disease (we defined individuals unsure of their prior history as eligible) and were currently receiving primary care services at North East Medical Services. Individuals were excluded if they were currently working as a healthcare worker or working in a hospital, nursing home, prison, correctional facility or homeless shelter (professions with occupational LTBI testing). Occupational testing and treatment were outside the scope of this study, as these individuals may have different perspectives, decisional context and may require distinct strategies to support LTBI care. Individuals were also excluded if they had previously taken LTBI treatment longer than a year ago. Although we were most interested in the preferences and perspectives of treatment-naïve individuals, we allowed inclusion of individuals with recent LTBI treatment to avoid bias as the clinic was in the process of expanding LTBI testing and treatment. Prior LTBI testing did not affect eligibility. Finally, we excluded individuals who were unable to complete the survey in English or in Chinese (simplified or traditional) or did not provide consent. All eligibility criteria were assessed based on self-report.

### Survey design

We ascertained preferences to treat LTBI in the context of various clinical, social and economic factors. An initial list of benefits, consequences of TB disease, as well as side effects from taking therapy, palatability of treatment, logistical burden of treatment and barriers related to LTBI treatment was generated based on a review of the literature and was refined and finalised by an interdisciplinary team consisting of public health researchers, epidemiologists, a health economic researcher, an attending physician at a pulmonary clinic, a primary care provider and community health workers serving this community as well as the directors of the local Departments of Public Health TB control programmes. We assessed participants’ hypothetical willingness to accept varying side effects, clinic visits, blood draws, drug–drug interactions, alcohol cessation, cost and reinfection risk, with the importance of each factor rated using a Likert scale. Survey questions were designed to inform the selection of attributes and levels for a larger choice-based study. In this context, estimating the proportion of participants willing to accept specific burdens was important to avoid including dominant levels and instead incorporate levels that participants might reasonably prefer to trade off (number of clinic visits or amount of out-of-pocket costs).

In addition, the survey also included a threshold exercise for 10-year TB progression risk, both to support future study design and because no prior work has assessed what TB progression risk individuals at risk of LTBI and TB consider meaningful. Participants were randomised to first see a 5% or 10% risk. The question was phrased as: ‘If 5 out of 100 people like you develop active TB disease in the next 10 years, would you prefer to take the treatment, or no treatment?’ or the equivalent for other risk probabilities. Based on the answer to the first question, the lowest or highest threshold was displayed next. The lowest threshold was labelled as ‘less than 1 out of 100’ and the highest was ‘50 out of 100’. Further risks were shown based on the participant’s answer, proceeding to smaller risks if the answer was ‘yes, prefer preventive treatment’ and higher risks if the answer was ‘no, prefer no treatment’, using the bisection method to limit the number of questions to five or fewer. All risks were displayed with an icon array (online supplemental figure S1).^26^

To rank different consequences of TB disease, participants first rated eight different consequences as not worrisome, moderately worrisome or very worrisome. In the next step, participants were asked to identify the top three most worrisome consequences among those they rated moderately or very worrisome, and then rank these three using a drag and drop interface. The questionnaire is included in online supplemental files materials 1 (English), 2 (traditional Chinese) and 3 (simplified Chinese).

We followed the Consensus-Based Checklist for Reporting of Survey Studies^27^ and included the checklist in online supplemental material 4.

### Procedures

The survey was conducted in the clinic’s lobby with the support of three community health workers who work at the community health centre. The study was advertised with a poster and table. Individuals approaching the table, which was staffed by community health workers, were consecutively recruited by age. We aimed to enrol 60 participants in total, with 20 participants in age groups 18–39, 40–59 and 60+ years. Participants received a $20 gift card for their participation. The survey was designed using the Qualtrics Survey Suite and was administered using an electronic tablet. Participants could complete the survey on their own or with one-on-one help from a trained, language-concordant community health worker in English, Mandarin or Cantonese for the entire survey or as needed. All survey responses were based on self-reported data. The survey included up to 54 questions on perspectives around LTBI treatment (with automatic skips for questions not applicable), 6 demographic questions and 4 feedback questions and took approximately 15 min to complete. Community health workers completed online training in human subjects research and received two 1-hour sessions of study-specific training on advertising and administering the survey, facilitated by two members of the research team.

The survey first elicited eligibility and provided basic information on TB, LTBI and preventive treatment for LTBI to ensure all participants had a standardised exposure to information before proceeding. Participants first ranked which consequences of TB disease would worry them the most. They then completed the threshold exercise, answered questions about potential barriers to LTBI treatment and finally completed demographic and socioeconomic information. To estimate opportunity cost—the value participants place on time spent receiving LTBI care at the clinic—we asked which other activities they would prefer instead of attending the clinic, and then how much they would be willing to pay to spend time on their preferred activities, using a slider scale (see questionnaire in online supplemental material 1). We randomised the order of presentation for multiple choice answers, including the consequences of TB disease, to minimise any ordering bias.

### Sample size

To describe the distribution of risk thresholds and how often other factors related to LTBI treatment could be potential barriers to treatment, we aimed for a total of 60 responses, which allowed for a frequency of 20% to be detected with a precision of ±10% for factors related to LTBI treatment.^28^ In addition, 60 responses would allow us to detect problems (eg, missing response options or inconsistencies) that occur in 5% of participants.^29^

### Patient and public involvement

Patients and the public were not involved in the conception and interpretation of the study. However, the study design was informed by qualitative literature on barriers related to LTBI treatment among migrants. This pilot study serves to assess individual perspectives and to inform the design of a larger, choice-based survey design to elicit patients’ preferences.

### Statistical analysis

The 10-year TB progression risk threshold at which persons would prefer LTBI treatment compared with no treatment was determined as the smallest risk at which the participant responded they would prefer treatment. We used descriptive statistics to report the remaining outcomes, including the ranking of TB consequences and the importance of different factors in the decision to take LTBI treatment or no treatment. We reported percentages calculated with a denominator of all participants, including those with missing answers, and reported the number of missing answers. Because there were few missing responses, no additional analyses were performed to address them. Similarly, we used descriptive analyses to summarise outcomes related to willingness to accept burden or harm when taking LTBI treatment such as varying side effects, clinic visits, blood draws, drug–drug interactions, cost and reinfection risk. In addition, for these outcomes, we classified different answers as potentially representing a barrier to LTBI care or favouring LTBI treatment (ie, compatible with current practice) based on the investigators’ clinical experience and knowledge of the health system. All analyses were performed in R V.4.1.2.

## Results

### Recruitment and participants

Study staff were approached by 64 individuals in the clinic lobby. Four were excluded: one was US-born, one was a healthcare worker, one individual did not consent (needed to go to an appointment) and one individual consented but did not complete the survey. Therefore, 60 individuals consented and completed the survey (completion rate 97% among 62 potentially eligible individuals).

Participants had a median age of 48 years (IQR 35–63 years); 52% were women and 100% spoke Chinese (table 1). Most participants were born in China, and participants immigrated a median of 15 years ago (IQR 7–23 years). 58 of 60 (97%) had health insurance. Many participants had little formal education; 52% did not complete high school or equivalent education. Two-thirds preferred not to report their household income; the majority of participants who answered reported a household income of less than US$25 000. Although some participants answered that they were unsure, none reported prior LTBI testing (no: n=47, 78%, unsure: n=13, 22%), prior LTBI treatment (no: n=57, 95%, unsure: n=3, 5%) or prior history of TB disease (no: n=57, 95%, unsure: n=3, 5%).

**Table 1:** Self-reported baseline characteristics of the 60 participants. For TB risk groups in the household, respondents could have multiple household members of different risk groups

	Number (percent) or median (**IQR**)
Female	31 (52%)
Age	48 (35–63)
Education	
No schooling	1 (2%)
Nursery school to grade 8	22 (37%)
Grade 9 or higher, without high school diploma	8 (13%)
Completed high school or equivalent	16 (27%)
Tertiary education	13 (22%)
Preferred language	
Chinese	60 (100%)
Country of birth	
China	42 (70%)
Hong Kong	7 (12%)
Vietnam	7 (12%)
Taiwan	4 (7%)
Years since immigration	15 (7–23)
Number of other people in household	2 (1–3)
TB risk groups in household	
Senior(s)	18 (30%)
Child/children	19 (32%)
Immunosuppressed person	0 (0%)
No risk group	21 (35%)
Missing	4 (7%)
Employment status	
Employed	28 (47%)
Self-employed	4 (7%)
Part-time	6 (10%)
Unemployed or seeking employment	3 (5%)
Student or further education	3 (5%)
Retired	10 (17%)
Prefer not to answer	6 (10%)
Annual household income	
Less than $25 000	17 (29%)
$25 001 to $50 000	1 (2%)
$50 001 to $75 000	3 (5%)
$75 001 to $100 000	0 (0%)
$100 001 to $150 000	1 (2%)
More than $150 000	0 (0%)
Prefer not to answer or missing	38 (63%)
Has health insurance	58 (97%)

Percentages may not add up to 100% due to rounding (education, country of birth, employment status and annual income).

TB, tuberculosis.

### Importance of different consequences of TB

Participants most often rated the risk of infecting others (n=52, 87%), the potential for isolation (n=44, 73%) and risk of death (n=42, 70%) as moderately or very worrisome consequences of TB disease (figure 1A). This pattern was consistent with a subsequent ranking exercise, where infecting others (n=35, 58%), risk of death (n=30, 50%) and potential isolation (n=25, 42%) were ranked as the top three most worrisome (figure 1B). Stigma (described as others could guess they are ill with TB) and requiring treatment for TB disease (with a 6-month standard treatment regimen) were the least worrisome consequences, ranked only by two participants (3%) as a top three worrisome consequence. Four participants (7%) rated all consequences as not worrisome, and 18 participants (30%) rated all consequences as either moderately or very worrisome.

**Figure 1:**
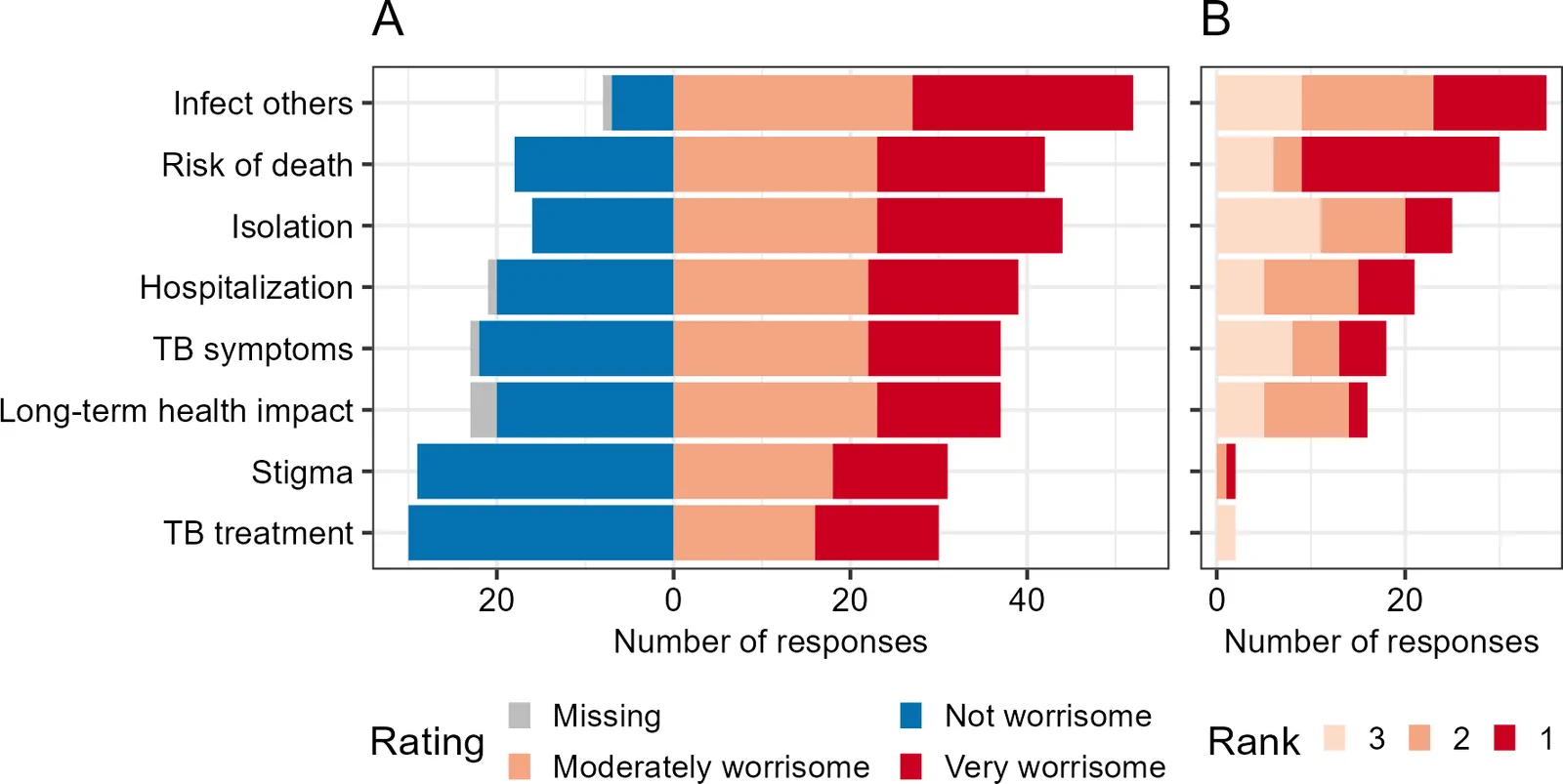
Participants rated, then ranked different consequences of tuberculosis. (**A**) Participants rated different consequences as not worrisome, moderately worrisome or very worrisome. (**B**) Participants selected the top three most worrisome consequences, with rank 1 being the most worrisome. TB, tuberculosis.

### Importance and acceptability of other factors

Figure 2 shows the ratings of the different factors related to the decision for or against LTBI treatment evaluated at the end of the survey. Reinfection risk (n=57, 95%), liver damage (n=57, 95%), cost or loss of income (n=56, 93%), TB progression risk (n=55, 92%), clinic visits (n=54, 90%) and blood draws (n=53, 88%) were most often rated as moderately or very important. Nausea or skin rash (n=46, 77%) and drug–drug interaction (n=44, 73%) were less often rated moderately or very important, followed by alcohol cessation (n=34, 57%). Eleven participants (18%) rated all factors for or against LTBI treatment equally, nine rated all as moderately worrisome and two rated all as very worrisome.

**Figure 2:**
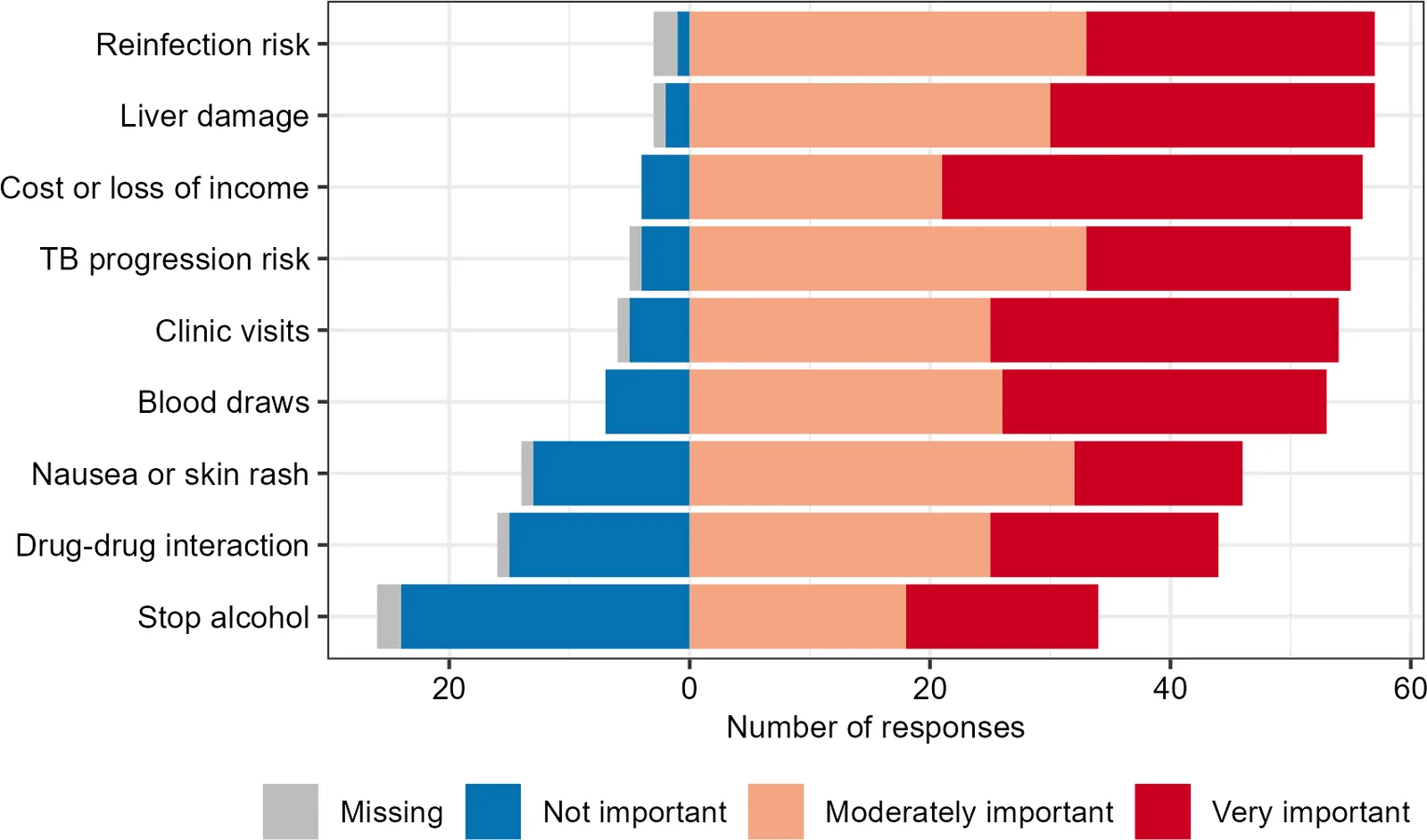
Importance of factors related to the decision for or against LTBI treatment rated on a Likert-type scale. Factors included potential barriers to treatment or burden of treatment. LTBI, latent tuberculosis infection; TB, tuberculosis.

### Thresholds of TB progression risk for LTBI treatment

Almost all participants (56 of 60, 93%) indicated that they were willing to take LTBI treatment even if their 10-year TB progression risk was less than 1% (figure 3). Three participants had risk thresholds of 3, 4 and 5%, respectively. One participant indicated not being willing to take LTBI treatment, even at a 50% 10-year TB progression risk without preventive treatment.

**Figure 3:**
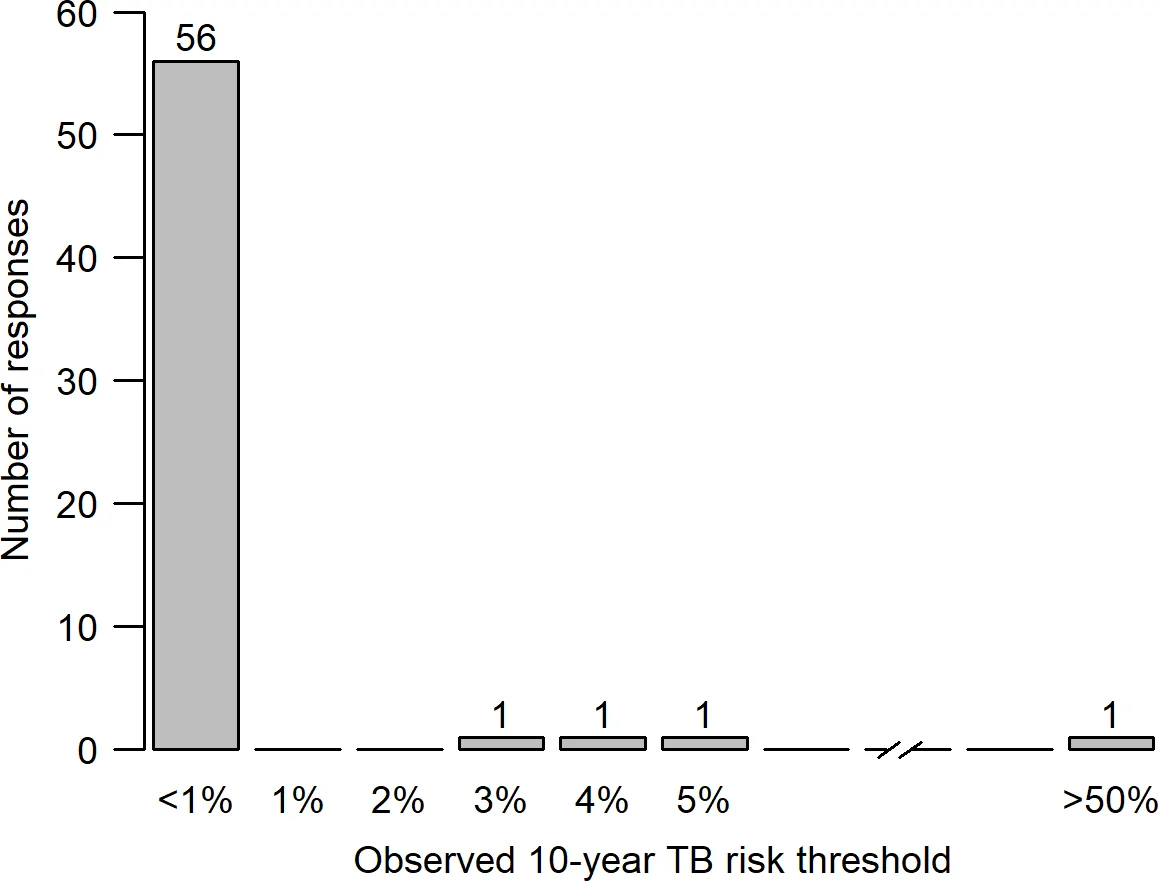
Observed tuberculosis (TB) risk thresholds after education on TB and brief education on preventive treatment.

We asked participants which time horizon (eg, 1 year, 2 years, 5 years, 10 years or lifetime risk) would be most important for when their doctor explains their progression risk. Most selected 1 year (n=24, 40%) or lifetime risk (n=14, 23%). Some selected 2 years (n=9, 15%) or 5 years (n=9, 15%), and only a few selected 10 years (n=2, 3%). One did not answer, and one did not select a time horizon but specified that LTBI treatment would very likely be prescribed (ie, the provider would recommend treatment, and they would follow the advice irrespective of risk or time horizon).

### Concern about reinfection risk

Most participants responded that they travel to countries where TB disease is common, either every year (n=8, 13%) or every 2–5 years (n=29, 48%). Only 7 (12%) did not anticipate travelling to any such country. After describing reinfection risk as about 1% among people who travel to countries where TB is common, most participants were at least slightly concerned (n=21, 35%) or reported being moderately concerned (n=12, 20%) or concerned (n=1, 2%). Less than half (n=26, 43%) were not concerned that they could ‘catch the TB germs again after completing preventive treatment, when traveling or for other reasons’. No one was extremely concerned.

### Willingness to accept burden, harm and cost for LTBI treatment

#### Cost and loss of income

Figure 4 summarises our findings ordered by how frequently factors favoured no LTBI treatment (shown in red and orange) and how often participants’ values were compatible with or favoured LTBI treatment (shown in blue). More than half of the participants were only willing to take LTBI treatment if it was free (n=32, 53%) and would only take LTBI treatment if they did not lose any income (n=36, 60%). Participants reported a median of $18 (IQR $0–100) as acceptable opportunity cost, described as the value of spending time on a preferred activity instead of seeking care for TB prevention.

**Figure 4:**
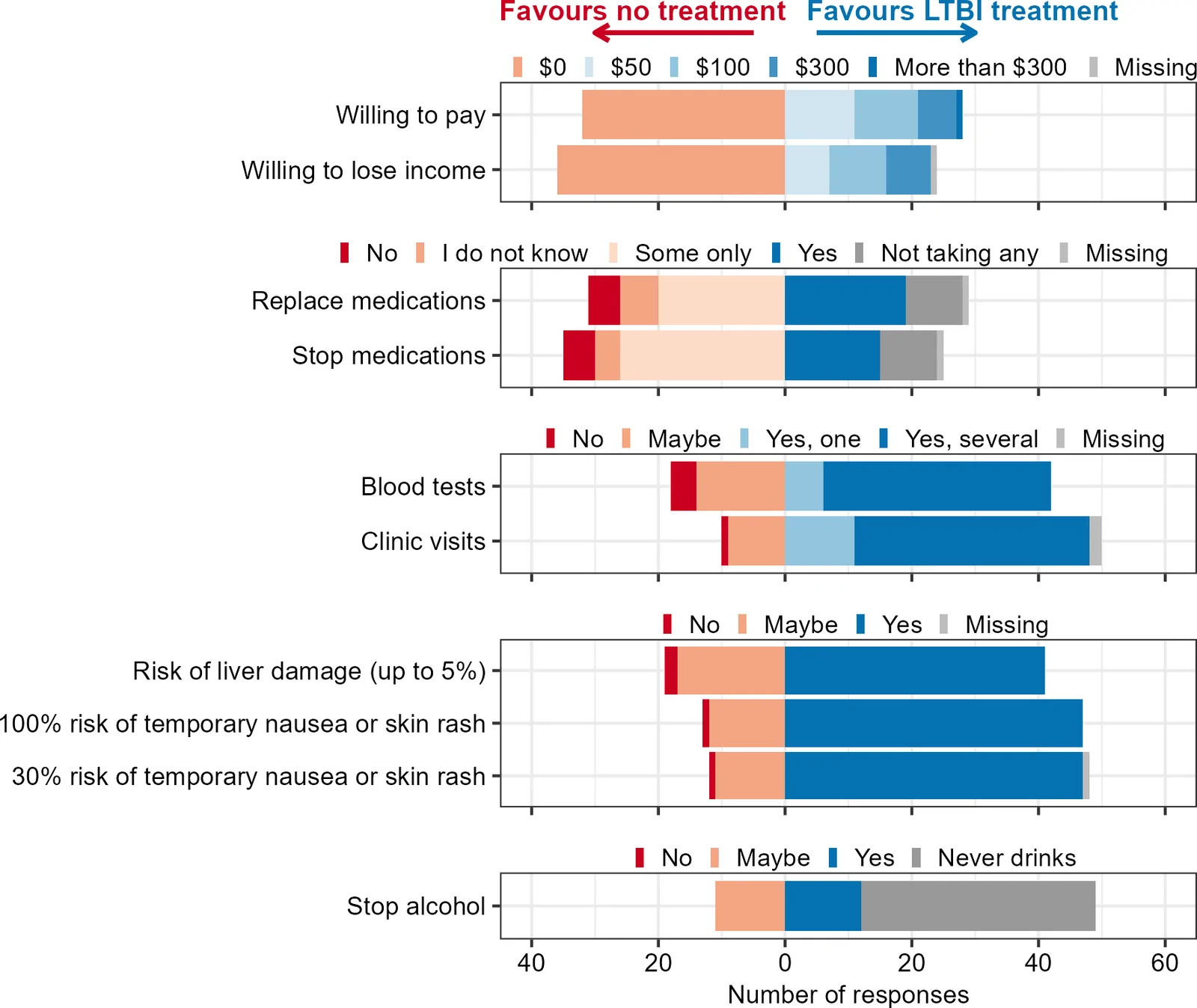
Number of participants with responses that favour LTBI treatment or favour no treatment (potential barriers). Answers that favour no LTBI treatment are shown in orange and red, answers that favour LTBI treatment, i.e., are compatible with taking treatment in the current health system, are shown in blue. Participants with missing answers or for whom the question did not apply are shown in grey. We assessed willingness to pay (out-of-pocket costs, travel costs or cost for child/elder care), willingness to accept loss of income, willingness to accept changes to other medications for a few months, willingness to accept monthly blood tests and several clinic visits, willingness to take LTBI treatment despite risk of adverse events and willingness to accept alcohol cessation for a few months, in order to take LTBI treatment. Monthly blood tests were presented as used to check if the liver and kidney are healthy, and LTBI treatment needs to be stopped. Clinic visits were presented as recommended to ensure the patient feels well and to help complete LTBI treatment. Temporary nausea or skin rash was described as one week, disappearing on their own without the need to see a doctor. Liver damage was described as requiring a hospital visit. LTBI, latent tuberculosis infection.

#### Clinic visits and blood tests

Most participants (n=36, 60%) reported they would accept monthly blood tests, described as ‘to make sure my liver and kidney are healthy’, although many remained unsure (n=14, 23%, shown as ‘maybe’ in figure 4). Others answered that they would only accept one blood test (n=6, 10%), or that they would take LTBI treatment but not accept blood tests (n=4, 7%).

Most participants answered that they would accept and attend several clinic visits (n=37, 64%) or one clinic visit (n=11, 19%) (figure 4). Only one participant answered they would not accept clinic visits. Among proposed visit types (in-person, by video or phone call) with their physician or pharmacists, two visit types were strongly preferred: 37 (63%) preferred an in-person visit with their physician, and 15 (25%) preferred a phone call with their physician. Less participants preferred a video call with their physician (n=3), or visits with their pharmacist (n=1 for in-person, n=1 for video and n=2 for phone call). One participant did not rank visit types. Participants who preferred a phone call were younger (median age 39 years) than participants who preferred an in-person visit (median age 59 years).

#### Drug–drug interactions

Despite comparatively low ratings of the importance of drug–drug interactions (figure 2), 51 of 60 participants (85%) reported taking medications: 31 took medications prescribed by their doctor, 9 took herbal or traditional medicine, 7 took over-the-counter medicine and 5 took hormonal contraception. Among participants who took medications, the majority were only willing to stop some (n=26, 51%) or none (n=5, 10%) of their medicines for a few months if they needed LTBI treatment. Similarly, most were only willing to replace some (n=20, 39%) or none (n=5, 10%) of their medicines (figure 4).

#### Adverse events

Liver damage was among the factors most frequently rated moderately or very important (n=57, 95%) in the decision for or against LTBI treatment (figure 2), whereas temporary nausea or skin rash was less often rated important (n=46, 77%). In a separate question, 20 participants (33%) reported worrying more about liver damage, 24 (40%) reported worrying about both liver damage and nausea or skin rash and 14 (23%) worried about neither liver damage nor nausea or skin rash, as preventing TB was more important. Only 2 (3%) were more worried about nausea or skin rash than liver damage. When asked whether ‘given the chance of liver damage (up to 5 in 100 people)’ participants would be willing to take LTBI treatment, 41 (68%) stated yes, 17 (28%) maybe and only 2 (3%) stated no. Similar answers were given for temporary nausea and skin rash (figure 4). Different risks of temporary nausea and skin rash did not meaningfully change participants’ willingness to take LTBI treatment.

#### Alcohol cessation

37 (62%) of the participants reported never drinking alcohol. Others reported drinking alcohol monthly or less (n=16, 27%), 2–4 times a month (n=5, 8%) and 2–3 times a week (n=2, 3%). No one reported drinking alcohol four or more times a week. Alcohol cessation was rated as the least important overall factor for deciding whether to accept LTBI treatment (figure 2). Among those who reported drinking alcohol, 12 (52%) reported being willing to stop alcohol for a few months and 11 (48%) answered maybe (figure 4).

## Discussion

In this pilot study among non-US-born individuals eligible for LTBI testing and receiving primary care at a large federally qualified health centre in the USA, we found a high willingness to take LTBI treatment even at low progression risks; however, participants indicated most potential harms or barriers to LTBI care that were presented to them were important. Notably, half of participants would not be willing to take LTBI treatment if there were any costs associated. This finding is especially relevant because while there is no cost-sharing for patients with Medicaid in our setting, patients may not be aware that treatment is free.^30^ In addition, there may be co-pays for visits or diagnostic tests^31^ and many patients face direct nonmedical costs such as travel or parking costs or opportunity costs such as loss of income for LTBI care.^15 17 32 33^ Although many participants did not report their annual household income, North East Medical Services primarily serves a low-income population, with about 60% of patients at or below the poverty level.^34^

In addition, the risk of reinfection after LTBI treatment was a common concern. A prior study found that some providers consider LTBI treatment not acceptable if there is a risk of reinfection,^17^ which may influence shared decision-making discussions. We also found that the risk of infecting others, risk of death, the frequency of hospitalisation and long-term health impact were the most worrisome consequences of progressing to TB disease. Thus, information brochures might consider content on the signs and symptoms of TB alongside consequences of TB disease such as hospitalisation, death and potential to infect others. Although isolation was one of the most worrisome consequences, guidance shortening isolation in community settings now exists.^35^ Similarly, although we described that a 6-month treatment would be necessary if LTBI progressed to TB disease, a 4-month treatment is now available for drug-susceptible TB.^22^ Overall, our findings emphasise that the decision to take LTBI treatment inherently requires patients in primary care to imagine a series of potential trade-offs, underscoring the importance of patient education and a shared decision-making approach.

Among the factors we considered for LTBI treatment decision-making, alcohol cessation, mild side effects like nausea and skin rash and drug–drug interactions were less important to participants. However, despite the comparatively low rating for the importance of drug–drug interactions, most participants reported taking other medications. Importantly, many were not willing to change or stop their medications. Therefore, drug–drug interactions and resulting medication changes may still represent a challenge to implementing TB prevention. The importance of drug–drug interactions to individuals has previously been shown for people living with HIV.^10 36^ Unfortunately, the shorter, preferred drug regimens contain rifamycins, which often interact with other drugs. Therefore, a regimen of 6 months of isoniazid is recommended for people unable to take a shorter preferred regimen due to drug intolerability or drug–drug interactions with rifamycins.^37^ Consistent with the overall importance rating, nausea and skin rash were considered less important than the risk of liver damage (a more severe side effect). This preference pattern is consistent with results from a study in Canada, where participants were more concerned about liver damage than skin rashes or fatigue,^9^ and a study in South Africa and one in Uganda, where side effects were least prioritised compared with other factors such as preventing disease.^10 38^ Liver damage may have been highly rated among individuals included in our sample, partly due to the importance of the liver in traditional Chinese medicine.^39^

Over 90% of participants were willing to take LTBI treatment even at a 10-year progression risk below 1%. The implication is that these participants would want treatment if they knew they were infected, regardless of their level of risk. These findings counter some providers’ perception that individuals do not want or prioritise LTBI care, as illustrated in one qualitative study.^17^ Prior preference studies in different settings that evaluated treatment choices at higher progression risks found variable acceptability: in Canada with an option for 9 months of isoniazid with a presented 10% risk over 10 years, 75% accepted LTBI treatment,^9 40^ whereas in Uganda with regimens of 1 or 3 months of isoniazid and rifapentine and a presented risk of 5% over 3 years,^10^ over 90% indicated they would accept treatment. In real-world clinical settings in the USA, however, treatment initiation rates are much lower.^24^ In addition, studies from high TB incidence settings showed that barriers to treatment uptake are reduced after TB education,^41^ and that the awareness of being at risk of TB increased treatment acceptance.^42^ Thus, our observed risk thresholds might have been low partly because participants were well-informed about TB and its consequences before completing the threshold exercise.

This study contributed to our understanding of preferences for LTBI treatment among a non-US-born Chinese-speaking population by including a detailed survey with high completion through self-administration or administration by a language-concordant community health worker. These steps allowed the broad inclusion of people of different ages and with less education. As we broadly assessed different factors for their relevance in the decisions around LTBI treatment, our findings can inform the design of a larger choice-based study, both for the selection of attributes and their levels. In particular, alcohol cessation could likely be omitted as an attribute for our population, as the majority never drank alcohol and no one was unwilling to stop drinking, whereas cost levels should not exceed $100, to allow meaningful trade-offs without cost becoming a dominant attribute.

The main limitations of our cross-sectional survey include the sample size (as this was only a pilot study), representativeness and hypothetical choices. Participants were not representative of all non-US-born populations, but were invited among a Chinese-speaking patient population in San Francisco with high prevalence of TB infection.^24^ Our survey measured hypothetical treatment preferences, and a larger choice-based study could better predict actual treatment choices.^40 43^ Furthermore, several factors could have contributed to bias in the high willingness to accept LTBI treatment even at a 10-year TB progression risk below 1%. First, when completing the threshold exercise, participants had only minimal prior information from the survey about LTBI treatment, which was described as 3–4 months of daily treatment with 1–2 antibiotics. We presented this task early in the survey, before discussing all potential barriers in detail, because we assumed it was the most cognitively demanding. Second, numeracy may have been low in this population, as many participants had little education. We used icon arrays to facilitate risk communication and address potential low numeracy.^26 44^ Third, administration by community health workers and recruitment in a healthcare setting may have influenced responses, potentially leading to social desirability bias or deference to a perceived provider recommendation. Therefore, it is possible that risk thresholds would be higher if participants had more information about the trade-offs, better understood small probabilities, were making the decision in a real-life context, or were recruited through different methods. Future studies should more explicitly evaluate the trade-offs between TB prevention and treatment burden using designs such as discrete choice experiments or conjoint analyses. Finally, the results of the rating exercise of all factors related to LTBI treatment should not be considered alone, as responses to other questions revealed that the importance could be underestimated (eg, changes to medications) or overestimated (eg, alcohol cessation among participants reporting no alcohol use). Few participants rated all factors equally worrisome, and ranking the factors may have helped gain more precise insights.

In conclusion, through this survey we found that among adults of all ages eligible for LTBI testing based on place of birth who receive primary care at a community health centre, the willingness to take LTBI treatment was high even in the context of low risk of progression, as long as testing and treatment are free. However, the benefit of treatment for avoiding progression to the harms of TB disease is likely often a trade-off with potential burdens or harms, including adverse events, clinic visits, blood draws and changes to other medications due to drug–drug interactions. Our findings can inform future studies that may measure the importance of these trade-offs with choice-based designs, ideally also considering whether the risk of reinfection matters to patients. Finally, our findings may inform person-centred decision-making tools for TB prevention in primary care settings.

## Supplementary material

10.1136/bmjopen-2026-121108 online supplemental file 1

10.1136/bmjopen-2026-121108 online supplemental file 2

10.1136/bmjopen-2026-121108 online supplemental file 3

10.1136/bmjopen-2026-121108 online supplemental file 4

10.1136/bmjopen-2026-121108 online supplemental figure 1

## Data Availability

Data are available upon reasonable request.
