# Supplementary material for: Preferences for treatment for latent tuberculosis infection in primary care among people in the USA at increased risk of tuberculosis: a pilot survey
Source: BMJ Open. 2026 Oct 14;16(10):e121108. doi: 10.1136/bmjopen-2026-121108 (PMC13691879; doi:10.1136/bmjopen-2026-121108)
Supplement: Supplementary file 1 [file bmjopen-2026-121108.reviewer_comments.pdf]

PEER REVIEW HISTORY

BMJ Open publishes all reviews undertaken for accepted manuscripts. Reviewers are asked to complete a checklist review form and are provided with free text boxes to elaborate on their assessment. These free text comments are reproduced below.

ARTICLE DETAILS

Title (Provisional)

Preferences for treatment for latent tuberculosis infection in primary care among people in the United States at increased risk of tuberculosis: a pilot survey

Authors

Aschmann, Hélène E.; Tang, Amy S.; Lee, Meagan; Salcedo, Katya L.; Murrill, Matthew T.; Chen, Gina; Ouyang, Yuqian; Lui, Kit; Rahman, Mehabuba; Flood, Jennifer; Kerkhoff, Andrew D; Lin, Tracy; Shete, Priya B; for the Tuberculosis Epidemiologic Studies Consortium, -

---

VERSION 1 - REVIEW

**Reviewer**	**1**
**Name**	**DeSilva, Malini B.**
**Affiliation**	**HealthPartners Institute for Medical Education, Research**
**Date**	**19-Jun-2026**
**COI**	**None**

This report describes a pilot survey fielded in a FQHC serving a primarily Chinese population to evaluate willingness to take LTBI treatment. While this is a pilot study, more could be added about the initial survey design and how the survey methods were chosen as well as culturally tailoring the survey along with the potential for expansion of the survey into other languages. A few additional considerations for revising the manuscript:

-Methods – although it is noted that 20 participants/age group were the initial aim, the sample size section describes why a total of 60 responses was chosen? Why this discrepancy?

-Lines 74 – 75 – Please revise this sentence, "The recommendation for treatment and completion of treatment have been reported as steps in the cascade with greater losses." Perhaps something like, 'Steps in the LTBI cascade with higher losses include recommending treatment and treatment completion."

-It would be helpful to report immigration status for the included participants as varying levels of TB testing occur based on immigration status

- Line 248 – TPT is listed for the first time, but not defined, please define TB preventive treatment or whatever definition is being used

- Line 319 – only a 4-month alternative is listed, however there are even shorter courses of LTBI treatment available now (3HP and 3HR)

-It seems that using the CONSORT extension checklist may be the most relevant reporting guideline to follow.

**Reviewer**	**2**
**Name**	**Chikovore, Jeremiah**
**Affiliation**	**Human Sciences Research Council**
**Date**	**13-Jul-2026**
**COI**	**None**

This manuscript reports a pilot survey exploring preferences regarding latent tuberculosis infection (LTBI) treatment among non-U.S.–born, predominantly Chinese-speaking primary care patients in San Francisco. The study addresses an important and underexplored area: patient-centered perspectives on LTBI treatment uptake, an essential component of TB elimination strategies. The focus on patient preferences, costs, reinfection concerns, and treatment trade-offs is timely and relevant. The high completion rate and inclusion of language-concordant community health workers are notable strengths. However, I have some comments and points of clarity for your consideration.

Abstract

1. Line 38, I suggest changing "Adults eligible for risk-based LTBI testing based on place of birth seeking primary care" to "Adults seeking primary care, eligible for risk-based LTBI testing based on place of birth"

2. The authors could more clearly spell the objective. As currently is, they highlight a gap, and then state they intend to address 'it', but the objective is not clearly spelt out – this is important in order to align also with outcome measures.

3. Authors also describe the design, but not the method specifically, hence it is not clear what was done to recruit the participants.

Body

1. Please state the current TB burden in the US. I would understand this might vary widely by socio-economic and demographic factors, and spatially. Nonetheless, this can provide some larger perspective.

2. Line 74-75: Suggest rewording to "Health worker recommendation for treatment and treatment completion have been reported as steps in the cascade with the greatest losses."

or "The stages of treatment recommendation and treatment completion have been reported as those with the greatest losses in the cascade."

3. Line 74-57"The term 'recommendation for treatment' is unclear as a cascade loss. Do the authors mean attrition among eligible individuals before receiving a treatment recommendation (for any reason, including loss to follow-up), a failure by health workers to offer or recommend treatment despite contact with an eligible individual, or a failure by individuals to take up a treatment recommendation once made? Please clarify the nature of this gap."

4. The text appears to place increasing emphasis on individual decision-making in relation to the feasibility of achieving TB elimination goals. It might be helpful to acknowledge in passing the substantial body of evidence demonstrating the influence of social, structural, health-system, and economic factors on engagement across the TB prevention and care cascade.

5. The statement 'the benefits of LTBI treatment (i.e., preventing TB)' appears somewhat restrictive. While prevention of active TB is the principal intended outcome, the benefits of LTBI treatment may extend beyond merely preventing TB disease itself; and because this broader framing does reflect in the rest of the paper, so authors may consider revisiting this statement.

6. “the benefits of LTBI treatment (i.e., preventing TB) may be limited in time by a risk of reinfection, particularly if individuals travel to countries with higher TB incidence” . It is unclear whether this statement refers to a factual limitation of LTBI treatment (i.e., the possibility of subsequent reinfection despite successful treatment). Clarification of this would be helpful.

7. The paragraph 81-90 needs to be pitched differently - emphasizing rather that patients perceive various challenges, (which affect uptake) and benefits (which facilitate uptake). If the paragraph is framed thus, it lays ground for the opening line in the next paragraph (Line 91)

8. In the main text, the statement 'To assess which of these factors and consequences exerts the most influence on LTBI treatment-related decisions, we conducted a pilot survey of non-U.S.–born persons' appears to function as a clear study objective. It would be helpful to ensure consistency between this objective and the objective presented in the abstract. The objectives under this paragraph (lines 91-97), including the purpose of the study, seem much more comprehensively describe; and could be replicated on summarised for abstract ti ensure consistency.

9. The rationale for excluding individuals employed in professions with occupational LTBI testing is understandable but could be stated, even briefly. The rationale for excluding individuals who had previously taken LTBI treatment more than one year ago is unclear.

10. The CDC determination that this activity constituted 'not research' - a brief explanation of the regulatory basis for this determination would improve transparency for readers.

11. The development of the preference schedule appears to be a critical component of the study, yet the description of its development is relatively brief. Given the complexity of identifying, selecting, and operationalizing factors that may influence LTBI treatment decisions, I would have expected a more detailed account of the instrument development process, including how domains were identified from the literature, how attributes were selected or prioritized, and how the final schedule was refined. I do understand this is a pilot, however, it would be helpful to comment on this aspect especially where instrument development is concerned. The manuscript notes the involvement of an interdisciplinary team, including a provider serving the target community and local public health directors, but it is unclear why these contributors are specifically highlighted. A fuller description of all individuals or stakeholder groups involved in the development and refinement of the preference schedule, and their respective roles, would strengthen confidence in the validity of the instrument.

12. The authors also note that they carried out a literature review as part of developing the tools. Was the review global, or restricted to US contexts?

13. Re: The survey first elicited eligibility and provided basic information on TB, LTBI, and preventive treatment for LTBI to ensure all participants had a standardized level of understanding before proceeding. While the survey may have provided participants with a standardized minimum exposure to information about TB, LTBI, and preventive treatment, it cannot be assumed that all participants subsequently attained a standardized level of understanding, given inevitable differences in health literacy, prior knowledge, comprehension, and engagement with the material. Consider revising this statement to indicate that the survey standardized participants' exposure to information rather than their understanding of it.

14. Line 152-54: Could the authors add some words to the sentence to ensure that it is understandable to a wider audience "Opportunity cost was assessed using a two-step question. Participants were first asked which activity they would have preferred to do instead of attending the clinic. They were then asked, using a slider scale, how much they would be willing to pay to spend time on that preferred activity rather than on the clinic visit. This amount was used as a measure of the value they placed on the time lost."

15. I miss a description of the time frame during which recruitment was carried out. Also, How much time did the entire survey take with a participant?

16. The eligibility criteria excluded individuals who had undergone LTBI testing more than one year previously. However, the manuscript later states (Line 192-193) that, while individuals with prior LTBI testing or recent LTBI treatment were eligible, no participants reported either. Can the authors clarify the rationale for the exclusion criterion related to

prior LTBI testing and explain how it aligns with the later statement. As presented, these statements appear difficult to reconcile.

17. Line 192-194: Participants were asked about prior LTBI testing, prior LTBI treatment, and prior TB disease. For each variable, please report the numbers responding "yes," "no," and "unsure," rather than only reporting the uncertain responses.

18. Please use consistent terminology when reporting survey responses. For example, "being contagious" and "infecting others" may not be interpreted identically by readers and could be perceived as measuring different concerns. Consistent wording would improve clarity and facilitate interpretation of the survey findings.

19. Please clarify how stigma was defined and assessed in the survey instrument. The phrase "others could guess they are ill with TB" appears to represent only one aspect of stigma. Additional detail would help readers understand what was actually measured and why.

20. Fairly high percentages of participants assigned identical ratings across all factors. Is there any other potential explanation(s) that could have implications for interpreting the findings?

21. Line 231-232: The survey question is clearer than the corresponding description in the manuscript. Consider rephrasing the text to indicate that participants were asked which TB risk prediction time horizon (1 year, 2 years, 5 years, 10 years, or lifetime) they considered most important when discussing their risk with a doctor.

22. A striking result is that 93% of participants reported willingness to accept LTBI treatment even at a <1% 10-year risk of progression. Could some of this be due to participants receiving educational information before the threshold exercise; questions being hypothetical and not linked to actual treatment decisions; community health workers administering some surveys; participants being recruited within a healthcare setting; treatment benefits possibly being emphasized more strongly than treatment burdens before the threshold exercise.). The discussion acknowledges and touches on some of these factors, but a more detailed reflection could also help.

23. The manuscript alternates between risk of developing TB; progression risk; 10-year TB risk. Consistent terminology would improve readability.

24. Over 60% of participants declined to report household income; how might this affect conclusions regarding cost sensitivity? It may also be helpful to reflect more on this high rate and what could explain it.

VERSION 1 - AUTHOR RESPONSE

<u>Reviewer 1:</u>

1. This report describes a pilot survey fielded in a FQHC serving a primarily Chinese population to evaluate willingness to take LTBI treatment. While this is a pilot study,

more could be added about the initial survey design and how the survey methods were chosen as well as culturally tailoring the survey along with the potential for expansion of the survey into other languages.

RESPONSE: Thank you for your careful review. We appreciate the opportunity to improve this manuscript.

Although we initially considered translating this survey also into Vietnamese and Spanish, only 2% of the patient population at this FQHC speak Vietnamese and 1% Spanish (see Tang et al. 2023, https://doi.org/10.1007/s10903-022-01438-1). Therefore, we decided to restrict the survey to English and Chinese.

To clarify the initial survey design and how the survey methods were chosen, we added the following information (lines 155-162):

> "*Survey questions were designed to inform the selection of attributes and levels for a larger choice-based study. In this context, estimating the proportion of participants willing to accept specific burdens was important to avoid including dominant levels and instead incorporate levels that participants might reasonably prefer to trade off (number of clinic visits or amount of out-of-pocket costs).*
> *In addition, the* survey also included a threshold exercise for 10-year TB progression risk*, both to support future study design and because no prior work has assessed what TB progression risk individuals at risk of LTBI and TB consider meaningful*."

Regarding cultural tailoring, the survey and its translation were reviewed by team members who are very familiar with this population, as well as a NEMS administrator who reviews all formal Chinese translations. Translations were provided in simplified and traditional Chinese. The NEMS team also provided feedback on the survey. Recruitment was also culturally tailored, as we had community health workers administer the survey both in Mandarin and Cantonese, and provide support for participants with limited literacy or vision impairment. We recruited in an area of the lobby that was heavily frequented by patients and with a set up that was familiar to the population that made them likely to engage with the community health workers at the survey table. These procedures are described in the methods of our manuscript (lines 179-180, and lines 184-186):

> "The survey was conducted in the clinic's lobby with the support of three community health workers who work at the community health center. The study was advertised with a poster and table."
> "Participants could complete the survey on their own or with one-on-one help from a trained, language-concordant community health worker in English, Mandarin, or Cantonese for the entire survey or as needed."

2. A few additional considerations for revising the manuscript:
   Methods – although it is noted that 20 participants/age group were the initial aim, the sample size section describes why a total of 60 responses was chosen? Why this discrepancy?
   RESPONSE: Thank you for the opportunity to clarify. The initial aim was 60 total responses to achieve sufficient precision to inform the design of a later choice-based study. We aimed to include 20 participants for each age group because we

hypothesized that these age groups may have different views or preferences, for example regarding the importance of drug-drug interactions with other medications, time spent for visits, or out-of-pocket costs. We have revised the wording as follows (lines 182-183):

> "*We aimed to enroll 60 participants in total with* 20 participants in age groups 18-39, 40-59, and 60+ years."

3. Lines 74 – 75 – Please revise this sentence, "The recommendation for treatment and completion of treatment have been reported as steps in the cascade with greater losses." Perhaps something like, 'Steps in the LTBI cascade with higher losses include recommending treatment and treatment completion."
RESPONSE: Thank you for this suggestion. We revised the sentence to (now lines 82-84):

> "*Steps in the LTBI care cascade with greater losses include treatment recommendation by providers and the treatment completion.*[4]"

4. It would be helpful to report immigration status for the included participants as varying levels of TB testing occur based on immigration status
RESPONSE: Although it is true that systematic TB testing depends on immigration status, unfortunately, we did not collect information on the immigration status beyond the year participants immigrated. This question was omitted because it was considered sensitive information and might have deterred participants from answering the survey. We also limited our reporting of the number of years since immigration to the interquartile range, as there were concerns in our team about reporting the full range.
Participants had immigrated a median of 15 years prior to the survey, with an interquartile range of 7 to 23 years (see Table 1).

5. Line 248 – TPT is listed for the first time, but not defined, please define TB preventive treatment or whatever definition is being used
RESPONSE: Thank you for catching this. We have replaced "TPT" with "LTBI treatment", as we refer to it as LTBI treatment throughout the manuscript (now line 302).

6. Line 319 – only a 4-month alternative is listed, however there are even shorter courses of LTBI treatment available now (3HP and 3HR)
RESPONSE: Thank you for the opportunity to clarify. Here we meant to explain that the standard treatment for active tuberculosis disease is 6-months, and we have described it as a 6-month treatment when we explained the consequences of tuberculosis disease to participants. However, nowadays a 4-month treatment is also available for drug-susceptible active tuberculosis disease.
To improve the readability of this sentence and distinguish more clearly between LTBI and TB disease, we have changed it to (lines 374-375):

> "Similarly, although we described *that* a 6-month treatment *would be necessary if LTBI progressed to TB disease*, a 4-month treatment is now available for drug-susceptible TB.[22]"

7. It seems that using the CONSORT extension checklist may be the most relevant reporting guideline to follow.

RESPONSE: Thank you for this suggestion. We had used the STROBE checklist (for observational studies) for our initial submission but were asked to provide the CROSS checklist (for survey studies) by the editor and agree with the editor that this is a better fit.

Reviewer 2:

This manuscript reports a pilot survey exploring preferences regarding latent tuberculosis infection (LTBI) treatment among non-U.S.–born, predominantly Chinese-speaking primary care patients in San Francisco. The study addresses an important and underexplored area: patient-centered perspectives on LTBI treatment uptake, an essential component of TB elimination strategies. The focus on patient preferences, costs, reinfection concerns, and treatment trade-offs is timely and relevant. The high completion rate and inclusion of language-concordant community health workers are notable strengths. However, I have some comments and points of clarity for your consideration.

RESPONSE: Thank you for your thoughtful and detailed review. We address your comments below and are grateful for the chance to improve this manuscript.

Abstract

1. Line 38, I suggest changing "Adults eligible for risk-based LTBI testing based on place of birth seeking primary care" to "Adults seeking primary care, eligible for risk-based LTBI testing based on place of birth"
   RESPONSE: Thank you, we have edited this sentence as you suggested.
2. The authors could more clearly spell the objective. As currently is, they highlight a gap, and then state they intend to address 'it', but the objective is not clearly spelt out – this is important in order to align also with outcome measures.
   RESPONSE: Thank you for pointing this out. We have revised the objective to (lines 31-34):

   > "*To understand which factors and perspectives around TB and LTBI treatment most influence individuals' willingness to take LTBI treatment*, we conducted a pilot preference survey among individuals from countries with high TB incidence."

   As you pointed out, this aligns better with the outcome measures, which are described as: "Perspectives on TB disease, risk of reinfection, and willingness to accept treatment if diagnosed with LTBI conditional on different factors, such as side effects, costs, and other treatment burden."
3. Authors also describe the design, but not the method specifically, hence it is not clear what was done to recruit the participants.
   RESPONSE: We have now specified that we used consecutive recruitment in the description of the setting (line 38):

   > "*Consecutive recruitment at a* federally qualified health center, serving a primarily Asian immigrant community, in San Francisco."

   Although this would exceed the level of detail we can include in the abstracts, to further clarify, our recruitment was conducted in the waiting area of the federally qualified health center. Patients of this center are used to advertisements displayed

on tables in this area, and it quickly attracted attention. We recruited consecutively until each age group had approximately 20 participants.

Body

1. Please state the current TB burden in the US. I would understand this might vary widely by socio-economic and demographic factors, and spatially. Nonetheless, this can provide some larger perspective.
   RESPONSE: We have added the following statement (lines 76-80):

   > "*Although the overall TB incidence rate in the United States is low (2.9 per 100 000 persons in 2023)*, non-U.S.–born communities are disproportionately affected by LTBI, with 76% of TB diagnoses in the United States occurring among these communities.[2] *Among non-U.S.–born Asian persons, the TB incidence rate is nearly eight times the overall national rate (22.5 per 100,000 person).[2]*"

2. Line 74-75: Suggest rewording to "Health worker recommendation for treatment and treatment completion have been reported as steps in the cascade with the greatest losses." or "The stages of treatment recommendation and treatment completion have been reported as those with the greatest losses in the cascade."
   RESPONSE: Thank you for this suggestion. As suggested by reviewer 1, we revised the sentence to (lines 82-84):

   > "Steps in the LTBI care cascade with greater losses include treatment recommendation by providers and the treatment completion.[4]"

3. Line 74-57"The term 'recommendation for treatment' is unclear as a cascade loss. Do the authors mean attrition among eligible individuals before receiving a treatment recommendation (for any reason, including loss to follow-up), a failure by health workers to offer or recommend treatment despite contact with an eligible individual, or a failure by individuals to take up a treatment recommendation once made? Please clarify the nature of this gap."
   RESPONSE: Thank you for the opportunity to clarify. We have now specified that this step in the cascade refers to providers recommending treatment (lines 82-84).

   > "Steps in the LTBI care cascade with greater losses include treatment recommendation *by providers* and the treatment completion.[4]"

   According to the systematic review by Alsdurf et al. (2016), the factors affecting the losses in this step are low provider knowledge about the need for LTBI treatment, medical contraindications for LTBI treatment (such as previous LTBI or TB treatment or liver disease), and social situations including substance abuse, recent release from jail or prison, lack of transportation, and fear of deportation or immigration status.

4. The text appears to place increasing emphasis on individual decision-making in relation to the feasibility of achieving TB elimination goals. It might be helpful to acknowledge in passing the substantial body of evidence demonstrating the influence of social, structural, health-system, and economic factors on engagement across the TB prevention and care cascade.
   RESPONSE: Thank you for pointing out this omission. We have added the following (lines 84-89):

"*Patient-level barriers to TB preventive care include limited healthcare access, lack of insurance coverage, transportation challenges, economic factors, fear of deportation, and low perceived risk of TB.*[4,5] *Provider-level barriers can include time pressures during clinic visits and knowledge gaps in how to test for and treat LTBI.*[4,6,7] *At the health system-level, competing priorities, chronic underfunding of primary care, and long wait times further hamper TB prevention.*[4,8]"

5. The statement 'the benefits of LTBI treatment (i.e., preventing TB)' appears somewhat restrictive. While prevention of active TB is the principal intended outcome, the benefits of LTBI treatment may extend beyond merely preventing TB disease itself; and because this broader framing does reflect in the rest of the paper, so authors may consider revisiting this statement.
RESPONSE: We have revised this to (lines 102-103):

"… the benefits of LTBI treatment (i.e., preventing TB *and its consequences*)"

We clarified the benefits of LTBI treatment further below in the same paragraph (lines 105-110):

"*Beyond reducing the risk of progressing to TB, LTBI treatment helps avoid the consequences of TB, including*: increased risk of death,[19] hospitalization,[20,21] significant symptoms, anticipated stigma, isolation, and lengthy treatment regimens of at least 4 or 6 months.[22] Finally, many TB survivors experience post-TB lung disease, reducing quality of life long-term and increasing mortality.[23]"

6. "the benefits of LTBI treatment (i.e., preventing TB) may be limited in time by a risk of reinfection, particularly if individuals travel to countries with higher TB incidence" . It is unclear whether this statement refers to a factual limitation of LTBI treatment (i.e., the possibility of subsequent reinfection despite successful treatment). Clarification of this would be helpful.
RESPONSE: Indeed, as you described, this referred to the fact that LTBI treatment is not necessarily protection against TB disease for an unlimited time (as a vaccine might be). Instead, if a reinfection occurs after successful treatment, LTBI treatment prevents TB disease only for the duration until the reinfection occurs. Provider interviews at the federally qualified health center have revealed that for some providers, the risk of reinfection was a major point in their counseling for or against LTBI treatment, particularly as many patients travel to China regularly. Others have found the same, as we have mentioned in the discussion section (lines 367-369):

"A prior study found that some providers consider LTBI treatment not acceptable if there is a risk of reinfection,[17] which may influence shared decision-making discussions."

We have used your wording to clarify that we mean risk of reinfection after successful treatment. This section was also further modified to address another reviewer's comment (lines 101-104).

"In addition, a challenge may be the risk of reinfection *after successful treatment*, as this may limit the benefits of LTBI treatment in time (i.e., preventing TB and its consequences). This may matter particularly to individuals planning to travel to countries with higher TB incidence.[14]"

7. The paragraph 81-90 needs to be pitched differently - emphasizing rather that patients perceive various challenges, (which affect uptake) and benefits (which facilitate uptake). If the paragraph is framed thus, it lays ground for the opening line in the next paragraph (Line 91)
   RESPONSE: Thank you for your helpful suggestion. We have revised the wording as you suggested (lines 97-112):

   > "*These barriers represent challenges that migrants may perceive regarding LTBI treatment and may affect uptake. These challenges include* taking pills, the duration of treatment, clinic visits and blood tests, as well as changes to other medications due to drug-drug interactions with rifamycins, willingness to cease alcohol consumption during the treatment, and potential costs of LTBI care, including loss of income and opportunity costs.[13,16,17] *In addition, a challenge may be the risk of reinfection after successful treatment, as this may limit the benefits of LTBI treatment in time* (i.e., preventing TB and its consequences). *This may matter particularly to individuals planning to* travel to countries with higher TB incidence.[18] On the other hand*, individuals may also perceive the benefits of LTBI treatment. Beyond reducing the risk of progressing to TB, LTBI treatment helps avoid* the consequences *of TB*, *including*: increased risk of death,[19] hospitalization,[20,21] significant symptoms, stigma, isolation, and *lengthy* treatment *regimens* of at least 4 or 6 months.[22] Finally, many TB survivors experience post-TB lung disease, reducing quality of life long-term and increasing mortality.[23]
   > To assess which of *these challenges and benefits exert* the most influence on LTBI treatment-related decisions…"

8. In the main text, the statement 'To assess which of these factors and consequences exerts the most influence on LTBI treatment-related decisions, we conducted a pilot survey of non-U.S.–born persons' appears to function as a clear study objective. It would be helpful to ensure consistency between this objective and the objective presented in the abstract. The objectives under this paragraph (lines 91-97), including the purpose of the study, seem much more comprehensively describe; and could be replicated on summarised for abstract ti ensure consistency.
   RESPONSE: Thank you for pointing this out. We believe our edited version to address your prior comment on the abstract now aligns well with the objective described at the end of the introduction section.

   Abstract, lines 31-34:

   > "*To understand which factors and perspectives around TB and LTBI treatment most influence individuals' willingness to take LTBI treatment*, we conducted a pilot preference survey among individuals from countries with high TB incidence."

   Introduction section, lines 111-113:

   > "To assess which of these *challenges* and *benefits exert* the most influence on LTBI treatment-related decisions, we conducted a pilot survey of non-U.S.–born persons eligible for LTBI testing."

9. The rationale for excluding individuals employed in professions with occupational LTBI testing is understandable but could be stated, even briefly. The rationale for

excluding individuals who had previously taken LTBI treatment more than one year ago is unclear.
RESPONSE: Thank you for the opportunity to clarify. As we were interested in individuals' perspectives and preferences who were eligible for LTBI treatment, treatment-naïve individuals were the most relevant for our purposes.

Individuals who have already undergone treatment may have different perspectives that may be shaped, for example, by their experiences of the health care system and potential side effects they may have experienced (or the lack of side effects). Thus, their willingness to take treatment could be higher or lower, depending on their individual experience.

However, we decided not to exclude individuals who had recently taken LTBI treatment, as the clinic where we recruited participants was scaling up their LTBI cascade. Therefore, if we had allowed only treatment-naïve participants, there was a risk that we might have excluded all individuals who were willing to take treatment, and surveyed only individuals who had already refused treatment.

We described this, and the reason for excluding individuals in professions with occupational LTBI testing, in the revised section on eligibility criteria (lines 129-136):

> "*Occupational testing and treatment were outside the scope of this study, as these individuals may have different perspectives, decisional context, and may require distinct strategies support LTBI care.* Individuals were also excluded if they had previously taken LTBI treatment longer than a year ago. *Although we were most interested in the preferences and perspectives of treatment-naïve individuals, we allowed inclusion of individuals with recent LTBI treatment to avoid bias as the clinic was in the process of expanding LTBI testing and treatment. Prior LTBI testing did not affect eligibility. Finally, we excluded individuals who were* unable to complete the survey in English or in Chinese (simplified or traditional) or did not provide consent."

10. The CDC determination that this activity constituted 'not research' - a brief explanation of the regulatory basis for this determination would improve transparency for readers.
RESPONSE: Thank you for pointing out this was unclear. We have revised the statement as shown below (lines 139-143 and lines 472-476):

> "The University of California San Francisco Institutional Review Board approved this study (#23-38710). All participants provided electronic consent. *The CDC received aggregate data of the study for intervention evaluation. Program evaluation of aggregate survey data of the survey* was reviewed by CDC, deemed not research, and conducted consistent with applicable federal law and CDC policy (see 45 C.F.R. part 46. 102 (l))."

Our survey was a sub-study conducted under the Tuberculosis Epidemiologic Studies Consortium. As such, the CDC funded the survey and received aggregate data. The main ethics review was performed by the UCSF IRB. In addition to this, the CDC only evaluated receiving aggregate data from the UCSF team. The CDC made a "not research – program evaluation" project determination for TBESC-III survey data given CDC received only aggregate data for the purpose of program evaluation. Accordingly, this was deemed not research – program evaluation.

11. The development of the preference schedule appears to be a critical component of the study, yet the description of its development is relatively brief. Given the complexity of identifying, selecting, and operationalizing factors that may influence LTBI treatment decisions, I would have expected a more detailed account of the instrument development process, including how domains were identified from the literature, how attributes were selected or prioritized, and how the final schedule was refined. I do understand this is a pilot, however, it would be helpful to comment on this aspect especially where instrument development is concerned. The manuscript notes the involvement of an interdisciplinary team, including a provider serving the target community and local public health directors, but it is unclear why these contributors are specifically highlighted. A fuller description of all individuals or stakeholder groups involved in the development and refinement of the preference schedule, and their respective roles, would strengthen confidence in the validity of the instrument.
    RESPONSE: Thank you for the opportunity to clarify. We have added the following explanation of how we selected the survey questions (lines 155-162):

    > "*Survey questions were designed to inform the selection of attributes and levels for a larger choice-based study. In this context, estimating the proportion of participants willing to accept specific burdens was important to avoid including dominant levels and instead incorporate levels that participants might reasonably to trade off (number of clinic visits or amount of out-of-pocket costs).*
    > *In addition, the* survey also included a threshold exercise for 10-year TB progression risk*, both to support future study design and because no prior work has assessed what TB progression risk individuals at risk of LTBI and TB consider meaningful*."

    In addition, we also clarified the roles and specialties of our interdisciplinary team (lines 149-152):

    "…was refined and finalized by an interdisciplinary *team consisting of public health researchers, epidemiologists, a health economic researcher, an attending physician at a pulmonary clinic,* a primary care provider *and community health workers* serving this community as well as the directors of the local Departments of Public Health TB control programs."

    The survey was refined iteratively in many aspects, including length, understandable and consistent lay language, as well as the order of questions. It was tested by the team prior to translation and recruitment of participants.

12. The authors also note that they carried out a literature review as part of developing the tools. Was the review global, or restricted to US contexts?
    RESPONSE: The literature review was global and not restricted to the US. We both searched for existing quantitative preference surveys, as well as qualitative studies that described the barriers and facilitators to accept and complete LTBI treatment, particularly among migrants. There were few quantitative studies, which mostly focused on regimen features, as described in lines 92-95:

    > "Although some preference studies compared regimen features in diverse settings,[9-12] little is known on how characteristics related to persons influence

preferences for accepting LTBI treatment, such as presumed progression risk or whether the person would need to modify or stop a current medication."

More importantly, barriers are described in lines 96-101 in the introduction section. Note that studies among migrants are usually conducted in countries with lower TB incidence.

> "Studies among migrants have identified multiple barriers for LTBI treatment initiation and adherence.[13-15] The burden of LTBI treatment includes taking pills, duration of treatment, clinic visits and blood tests, as well as changes to other medications due to drug-drug interactions with rifamycins, willingness to cease alcohol consumption during the treatment, and potential costs of LTBI care, including loss of income and opportunity costs.[13,16,17]"

13. Re: The survey first elicited eligibility and provided basic information on TB, LTBI, and preventive treatment for LTBI to ensure all participants had a standardized level of understanding before proceeding. While the survey may have provided participants with a standardized minimum exposure to information about TB, LTBI, and preventive treatment, it cannot be assumed that all participants subsequently attained a standardized level of understanding, given inevitable differences in health literacy, prior knowledge, comprehension, and engagement with the material. Consider revising this statement to indicate that the survey standardized participants' exposure to information rather than their understanding of it.
RESPONSE: Thank you for pointing this out, this is an important distinction. We have edited the text accordingly (lines 193-195):

> "The survey first elicited eligibility and provided basic information on TB, LTBI, and preventive treatment for LTBI to ensure all participants had a *standardized exposure to information* before proceeding."

14. Line 152-54: Could the authors add some words to the sentence to ensure that it is understandable to a wider audience "Opportunity cost was assessed using a two-step question. Participants were first asked which activity they would have preferred to do instead of attending the clinic. They were then asked, using a slider scale, how much they would be willing to pay to spend time on that preferred activity rather than on the clinic visit. This amount was used as a measure of the value they placed on the time lost."
RESPONSE: Thank you for the opportunity to clarify. We have modified the sentence in the following manner (lines 197-202):

> "*To estimate opportunity cost – the value participants place on the time spent receiving LTBI care at the clinic – we asked* which other activities they would prefer *instead of attending the clinic, and then how much they would be willing to pay* to spend time on their preferred activities, *using a slider scale* (see questionnaire in online supplemental material 1)."

We hope that with the addition of an explanation of what opportunity cost is, this sentence will be understandable to a wider, non-economic audience.

15. I miss a description of the time frame during which recruitment was carried out. Also, How much time did the entire survey take with a participant?
RESPONSE: Thank you for this question. We mentioned the time frame of the recruitment in the first sentence of the methods (line 121):

> "We conducted a cross-sectional pilot survey among individuals eligible for LTBI testing in October 2023."

To be exact, the recruitment took place from October 12, 2023 to October 30, 2023.

The survey was set up to automatically capture the time taken to complete the survey. Unfortunately, the timer started as the community health workers re-opened the survey on an electronic tablet in preparation of recruiting the next participant, and not when the participant answered the first question. Therefore, no accurate timing was captured. Although the analysis suggests that the median time was 19 minutes with an interquartile range of 15 to 25 minutes, the community health care workers reported that most surveys took about 15 minutes. All these time estimates include eligibility screening and informed consent. We included the following in the manuscript (lines 187-192):

> "*The survey included up to 54 questions on perspectives around LTBI treatment (with automatic skips for questions not applicable), 6 demographic questions, 4 feedback questions, and took approximately 15 minutes to complete.*"

We included the number of questions because it is suggested in the CROSS checklist.

16. The eligibility criteria excluded individuals who had undergone LTBI testing more than one year previously. However, the manuscript later states (Line 192-193) that, while individuals with prior LTBI testing or recent LTBI treatment were eligible, no participants reported either. Can the authors clarify the rationale for the exclusion criterion related to prior LTBI testing and explain how it aligns with the later statement. As presented, these statements appear difficult to reconcile.
RESPONSE: Thank you for the opportunity to clarify. Only LTBI treatment longer than a year ago was an exclusion criterion. History of LTBI testing had no impact on eligibility. We have added the following sentence to the description of the eligibility criteria (lines 134-135):

> "*Prior LTBI testing did not affect eligibility.*"

To avoid further confusion, we have also modified the text (now lines 240-242), and removed the reference to eligibility criteria:

> "*Although some participants answered that they were unsure, none reported prior LTBI testing (no: n=47, 78%, unsure: n=13, 22%), prior LTBI treatment (no: n=57, 95%, unsure: n=3, 5%), or prior history of TB disease (no: n=57, 95%, unsure: n=3, 5%).*"

17. Line 192-194: Participants were asked about prior LTBI testing, prior LTBI treatment, and prior TB disease. For each variable, please report the numbers responding "yes," "no," and "unsure," rather than only reporting the uncertain responses.
RESPONSE: Thank you for this valuable suggestion. We have added the number of answers for "no". However, since "yes" was always zero, we have omitted it for simplicity (now lines 240-242).

> "*Although some participants answered that they were unsure, none reported prior LTBI testing (no: n=47, 78%, unsure: n=13, 22%), prior LTBI treatment*

(no: n=57, 95%, unsure: n=3, 5%), or prior history of TB disease (no: n=57, 95%, unsure: n=3, 5%)."

18. Please use consistent terminology when reporting survey responses. For example, "being contagious" and "infecting others" may not be interpreted identically by readers and could be perceived as measuring different concerns. Consistent wording would improve clarity and facilitate interpretation of the survey findings.
    RESPONSE: Thank you for catching this. We have revised the wording of "being contagious" to "the risk of infecting others" (line 252):

    > "Participants most often rated *the risk of infecting others* (n=52, 87%), the potential for isolation (n=44, 73%), and risk of death (n=42, 70%) as moderately or very worrisome consequences of TB disease (Figure 1A)."

19. Please clarify how stigma was defined and assessed in the survey instrument. The phrase "others could guess they are ill with TB" appears to represent only one aspect of stigma. Additional detail would help readers understand what was actually measured and why.
    RESPONSE: Thank you for the opportunity to clarify. It is true that tuberculosis can come with diverse forms and aspects of stigma, including fear of contagion, social exclusion, blame for illness, association with poverty or HIV. We described only the anticipated stigma of others guessing an individual is ill with TB, as further stigma depends on this. We have made small modifications to the wording in the manuscript, including the specification of "anticipated stigma" in line 108, and in the explanation of figure 1 (line 256):

    > "Stigma (*described as* others could guess they are ill with TB) …"

    The association with HIV is an important source of stigma in other settings such as Sub-Saharan Africa. In our setting, the association with poverty is more likely.

20. Fairly high percentages of participants assigned identical ratings across all factors. Is there any other potential explanation(s) that could have implications for interpreting the findings?
    RESPONSE: Thank you for this question. We reported the proportion of participants who assigned identical ratings for both the consequences of TB (figure 1), as well as the factors presented in figure 2.

    In general, in rating exercises, it is commonly observed that participants do not distinguish well between the presented options. Ranking exercises are better suited to separate out what matters more to individuals, among options that all matter to them. Nevertheless, rating exercises clarify whether these options matter to individuals at all.
    This is why, for the consequences of TB, we used a ranking exercise after rating the consequences. Here, one of our goals was to find out what information was most crucial to present on TB disease if we had limited time. We still reported the ratings, because it was somewhat surprising, for example, that any participants would perceive all of the described consequences of TB as not worrisome.
    We did not include a ranking exercise for the factors presented in figure 2, as this seemed cognitively too demanding and needed to be at the end of the survey. Instead, community health care workers took special care to emphasize the importance of the rating exercise presented in figure 2. We believe this helped to limit

so-called straight-lining to what we deem surprisingly few participants – only 11 out of 60. We have added the following limitation in the discussion (439-440):

> "Few participants rated all factors equally worrisome, and ranking the factors may have helped gain more precise insights."

21. Line 231-232: The survey question is clearer than the corresponding description in the manuscript. Consider rephrasing the text to indicate that participants were asked which TB risk prediction time horizon (1 year, 2 years, 5 years, 10 years, or lifetime) they considered most important when discussing their risk with a doctor.
RESPONSE: Thank you for this suggestion to improve the clarity of the manuscript. We have rephrased it as (lines 283-284):

> "We asked participants *which time horizon (e.g., 1 year, 2 years, 5 years, 10 years, or lifetime risk)* would be most important for when their doctor *explains their progression risk*."

22. A striking result is that 93% of participants reported willingness to accept LTBI treatment even at a <1% 10-year risk of progression. Could some of this be due to participants receiving educational information before the threshold exercise; questions being hypothetical and not linked to actual treatment decisions; community health workers administering some surveys; participants being recruited within a healthcare setting; treatment benefits possibly being emphasized more strongly than treatment burdens before the threshold exercise.). The discussion acknowledges and touches on some of these factors, but a more detailed reflection could also help.
RESPONSE: Thank you for emphasizing this point. We agree with all of these points and have restructured and added to the limitation section to better clarify these factors (lines 422-439).

> "*Furthermore, several factors could have contributed to bias in the high willingness to accept LTBI treatment even at a 10-year TB progression risk below 1%. First,* when completing the threshold exercise, participants had only minimal prior information from the survey about LTBI treatment, which was described as 3-4 months of daily treatment with 1-2 antibiotics. We presented this task early in the survey, before discussing all potential barriers in detail, because we assumed it was the most cognitively demanding. *Second*, numeracy may *have been* low in this population, as many participants had little education. We used icon arrays to facilitate risk communication and address potential low numeracy.[26,44] *Third*, administration by community health workers *and recruitment in a health-care setting may have influenced* responses, *potentially leading* to social desirability bias *or deference to a perceived provider recommendation*. Therefore, it is possible that risk thresholds would be higher if participants *had more information* about the trade-offs, *better understood small probabilities, were making the decision in a real-life context, or were recruited through different methods*. Future studies should *more explicitly* evaluate the trade-offs between TB prevention and treatment burden using designs such as discrete choice experiments or conjoint analyses."

23. The manuscript alternates between risk of developing TB; progression risk; 10-year TB risk. Consistent terminology would improve readability.

RESPONSE: Thank you for pointing this out. We have changed this to using only "progression risk" or "risk of progressing to TB", and where the time horizon was relevant, "10-year TB progression risk". There remains only one instance of "developing TB disease" where we quoted from the survey instrument.

24. Over 60% of participants declined to report household income; how might this affect conclusions regarding cost sensitivity? It may also be helpful to reflect more on this high rate and what could explain it.
    RESPONSE: It is most likely that most participants had low household income. This federally qualified health center primarily serves the Chinese population in a deprived neighborhood and has special plans and discounts for individuals with low income. Based on the NEMS 2025 UDS report, 59.2% of NEMS patients are at 100% and below the poverty level, 16.1% are 101-150% above the poverty level, 7.9% at 151-200% above the poverty level, and 16.7% more than 200% over the poverty level.
    In addition, if insurance is considered a surrogate for income levels, 73.8% NEMS patients have Medicaid and 7.9% are uninsured.
    To clarify this, we have added the following in the discussion (lines 364-266):

    > "*Although many participants did not report their annual household income, NEMS primarily serves a low-income population, with about 60% of patients at or below the poverty level*.[34]"
